# Expression of the Lantibiotic Mersacidin in *Bacillus amyloliquefaciens* FZB42

**DOI:** 10.1371/journal.pone.0022389

**Published:** 2011-07-21

**Authors:** Anna Maria Herzner, Jasmin Dischinger, Christiane Szekat, Michaele Josten, Stephanie Schmitz, Anja Yakéléba, Ricarda Reinartz, Andrea Jansen, Hans-Georg Sahl, Jörn Piel, Gabriele Bierbaum

**Affiliations:** 1 Institute of Medical Microbiology, Immunology and Parasitology, University of Bonn, Bonn, Germany; 2 Kekulé Institute of Organic Chemistry and Biochemistry, University of Bonn, Bonn, Germany; University of Groningen, The Netherlands

## Abstract

Lantibiotics are small peptide antibiotics that contain the characteristic thioether amino acids lanthionine and methyllanthionine. As ribosomally synthesized peptides, lantibiotics possess biosynthetic gene clusters which contain the structural gene (*lanA*) as well as the other genes which are involved in lantibiotic modification (*lanM*, *lanB*, *lanC, lanP*), regulation (*lanR*, *lanK*), export (*lanT(P)*) and immunity (*lanEFG*). The lantibiotic mersacidin is produced by *Bacillus* sp. HIL Y-85,54728, which is not naturally competent.

**Methodology/Principal Findings:**

The aim of these studies was to test if the production of mersacidin could be transferred to a naturally competent *Bacillus* strain employing genomic DNA of the producer strain. *Bacillus amyloliquefaciens* FZB42 was chosen for these experiments because it already harbors the mersacidin immunity genes. After transfer of the biosynthetic part of the gene cluster by competence transformation, production of active mersacidin was obtained from a plasmid *in trans*. Furthermore, comparison of several DNA sequences and biochemical testing of *B. amyloliquefaciens* FZB42 and *B.* sp. HIL Y-85,54728 showed that the producer strain of mersacidin is a member of the species *B. amyloliquefaciens*.

**Conclusions/Significance:**

The lantibiotic mersacidin can be produced in *B. amyloliquefaciens* FZB42, which is closely related to the wild type producer strain of mersacidin. The new mersacidin producer strain enables us to use the full potential of the biosynthetic gene cluster for genetic manipulation and downstream modification approaches.

## Introduction

The lantibiotic (i.e. lanthionine-containing antibiotic) mersacidin is an antimicrobial peptide that consists of 20 amino acids. The producer strain of mersacidin, *Bacillus* sp. HIL Y-85,54728 [1], has not yet been closely characterized. The structural gene (*mrsA*) and the genes for modification enzymes, transporters and producer self-protection are encoded on a 12.3 kb biosynthetic gene cluster on the chromosome of the producer strain [2]. Moreover, three regulatory genes are present in the gene cluster. The two-component regulatory system MrsR2/K2 is mainly involved in immunity and induction of mersacidin biosynthesis in the presence of mersacidin by a quorum sensing mechanism [3]. A further single regulatory protein, MrsR1, is encoded downstream of *mrsA* and is essential for mersacidin production [4]. Mersacidin inhibits the growth of gram-positive bacteria by binding to the cell wall precursor lipid II and thereby inhibiting cell wall biosynthesis [5].

Production of mersacidin and genetically engineered mersacidin peptides has so far been performed in variants of the original producer strain, *Bacillus* sp. HIL Y-85,54728. Production of engineered peptides was obtained either *in trans* after inactivation of *mrsA* by introduction of a stop codon [6] or *in cis* after double homologous recombination [7]. However, transformation of the producer strain with exogenous plasmids could only be achieved by protoplast transformation or electroporation and both methods yielded only low transformation frequencies. Therefore, the aim of these studies was to build an expression system for mersacidin in a naturally competent *Bacillus* strain in synthetic medium and exploit competence transformation as an efficient method for the transfer of the biosynthetic gene cluster and plasmids harboring *mrsA*.

Successful heterologous production has previously been shown for several lantibiotics, e. g. subtilin and nisin by *B. subtilis* 168 [8], [9], lacticin 3147 by *Enterococcus faecalis* [10], or epicidin 280 by *Staphylococcus carnosus* [11]. Very recently, production of active lichenicidin has even been achieved in *Escherichia coli* [12]. However, heterologous production of a lantibiotic cannot be taken for granted and remains difficult. For example, epicidin 280 and Pep5 are two closely related lantibiotics, but Pep5 shows a higher antibacterial activity than epicidin 280. In contrast to epicidin 280 [11], Pep5 cannot be expressed in *S. carnosus*, which is susceptible to this agent in the nanomolar range, indicating that the toxicity of the product may be a problem here (G. Bierbaum, unpublished data). Therefore, for successful and high-level expression of mersacidin in a heterologous host, two conditions had to be met, functional producer self-protection and transfer of the biosynthetic gene cluster to the new host. The genome of *Bacillus amyloliquefaciens* FZB42, a competent plant-growth promoting rhizobacterium, has recently been sequenced. The part of the mersacidin biosynthetic gene cluster, that is devoted to producer self-protection, is already present in this organism [13] making it an ideal candidate for the transfer of the biosynthetic genes. Here we show that production of mersacidin is possible in *B. amyloliquefaciens* FZB42 and that the mersacidin producer strain itself is a member of this species.

## Results and Discussion

### *B. amyloliquefaciens* FZB42 is immune to mersacidin

**Figure 1:**
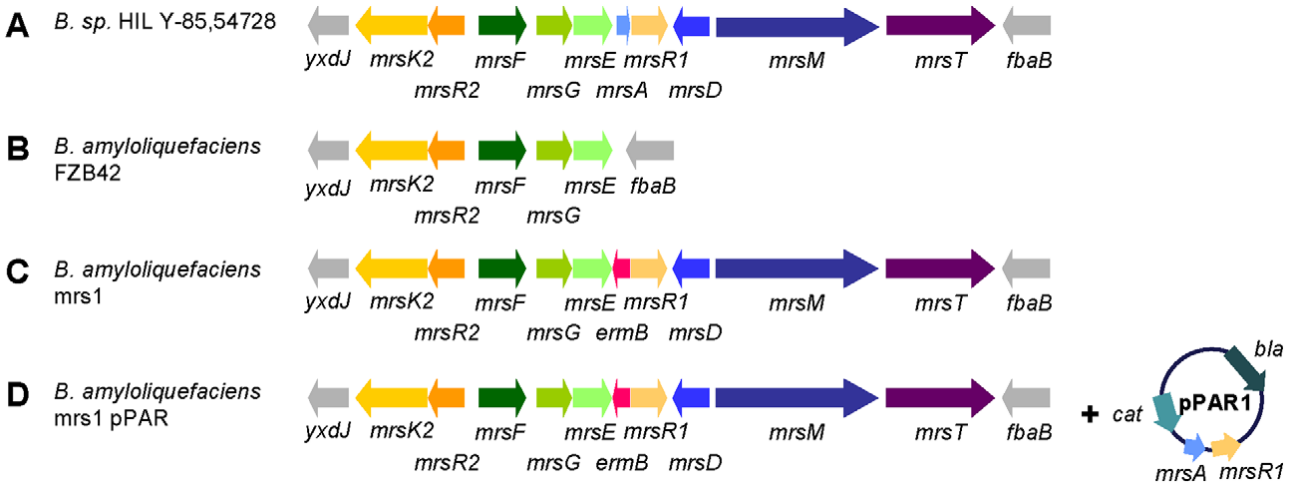
The (partial) mersacidin biosynthesis gene clusters of the mersacidin producer *B.* sp. HIL Y-85,54728, *B. amyloliquefaciens* FZB42 and its derivatives. The mersacidin gene cluster of the original producer strain *B.* sp. HIL Y-85,54728 (**A**) consists of the immunity genes *mrsFGE* (green colors), the structural gene *mrsA* (light blue), the modification enzymes *mrsD* and *mrsM* (dark blue colors), the exporter containing a protease domain *mrsT* (purple) and the regulatory genes *mrsR1, mrsR2* and *mrsK2* (yellow and orange colors). The genome of *B. amyloliquefaciens* FZB42 (**B**) harbors a partial mersacidin gene cluster consisting of the immunity genes *mrsFGE* and the regulatory genes *mrsK2* and *mrsR2*. The genes are found at the same site as in the original producer strain, i. e. between *ycdJ* and *fbaB*. In the mutant strain *B. amyloliquefaciens* mrs1 (**C**), a partial completion of the mersacidin gene cluster was reached by competence transformation using genomic DNA of a mersacidin deletion mutant (*B*. sp. HIL Y-85,54728 Rec1). An erythromycin resistance (*ermB*) cassette substituting *mrsA* served as selection marker. *mrsR1* is most probably not transcribed in this mutant because of a polar effect. The completion of the mersacidin gene cluster in *B. amyloliquefaciens* mrs1 pPAR1 (**D**) was achieved *in trans* by transformation with the plasmid pPAR1, carrying the structural gene *mrsA* and *mrsR1*, yielding *B. amyloliquefaciens* mrs1 pPAR1.

Analysis of the complete genome sequence of *B. amyloliquefaciens* FZB42 [13] had shown, that *mrsFGE*, which encode an ABC transporter that inhibits binding of mersacidin to the cells [4], and *mrsK2R2*, the genes of a two component system that induces expression of *mrsFGE* in the presence of mersacidin [3], are present in this organism (Fig. 1). These genes are located at the same site as in the original producer strain of mersacidin, i. e. between *ycdJ* and *fbaB* [2]. A detailed comparison showed that the encoded proteins share at least 98 % amino acid identity (two exchanges in MrsE and MrsF, one exchange in MrsR2 and four exchanges in MrsK2), with the exception of the N-terminus of MrsG. However, resequencing of the wild type producer indicated that a thymidine residue in position 980 was missing in the original sequence of *mrsG*. After sequence correction, the MrsG amino acid sequences were identical. In the intergenic region between *mrsE* and *fbaB*, a short sequence of 147 bp is inserted in the genome of *B. amyloliquefaciens* FZB42 (bp 3774591 to 3774738) that is not present in the producer strain of mersacidin and did not yield any hits in the databases. This sequence is flanked by two distinct regions with sequence similarity to the mersacidin gene cluster. The upstream 38 bp region is similar to the sequence found downstream of *mrsE*, i. e. the sequence upstream of the putative *mrsA* operator. The 89 bp region found downstream of the 147 bp insert is homologous to the sequence downstream of *mrsT*, contains the inverted repeat that is thought to delimit the mersacidin biosynthetic gene cluster and is not present in *B. amyloliquefaciens* DSM 7^T^. This might indicate that the biosynthetic part of the gene cluster was lost from *B. amyloliquefaciens* FZB42 during evolution.

A previous comparison of minimum inhibitory concentration (MIC) values of the wild type producer and an *mrsK2R2* knockout clone, which does not express the mersacidin immunity genes *mrsFGE*, had demonstrated that expression of *mrsK2R2FGE* increased the resistance to mersacidin about threefold [4]. MIC determinations of the wild type producer (25 mg/l) and *B. amyloliquefaciens* FZB42 (25 mg/l) demonstrated that *B. amyloliquefaciens* FZB42 was at least as resistant to mersacidin as the producer strain and therefore this organism was chosen as amenable to mersacidin production.

### Reconstitution of mersacidin production in *B. amyloliquefaciens* FZB42

In order to transfer the biosynthetic part of the mersacidin biosynthetic gene cluster into *B. amyloliquefaciens* FZB42, chromosomal DNA of *Bacillus* sp. HIL Y-85,54728 Rec1 was utilized. This strain harbors the complete mersacidin gene cluster including the operator sequence, apart from *mrsA* and its promoter that have been replaced by an erythromycin resistance cassette [2]. Therefore, the use of erythromycin as a selection marker for successful integration of the gene cluster was possible during transformation experiments. After a competence transformation, 15 erythromycin resistant colonies were isolated and insertion of the biosynthetic part of the mersacidin biosynthesis gene cluster was confirmed by PCR, employing the primers mrsE681.f and ermB665.r annealing in *mrsE* and *ermB* as well as mrsT2251.f and fbaB283.r annealing in *mrsT* and *fabB*. A PCR of *comK*, employing primers located in the intergenic region (FZBfor, FZBrev) that did not match the sequence of the wild type producer strain confirmed that the clones were indeed *B. amyloliquefaciens* FZB42 transformants and did not derive from spores of the producer strain. Subsequently, one of these clones, *B. amyloliquefaciens* FZB42 mrs1, was transformed with pPAR1 [3], which harbors *mrsA* and *mrsR1*, by competence transformation. The regulator MrsR1 is essential for mersacidin biosynthesis [4] and is transcribed from the *mrsA* promoter (Bierbaum, unpublished results).

**Figure 2:**
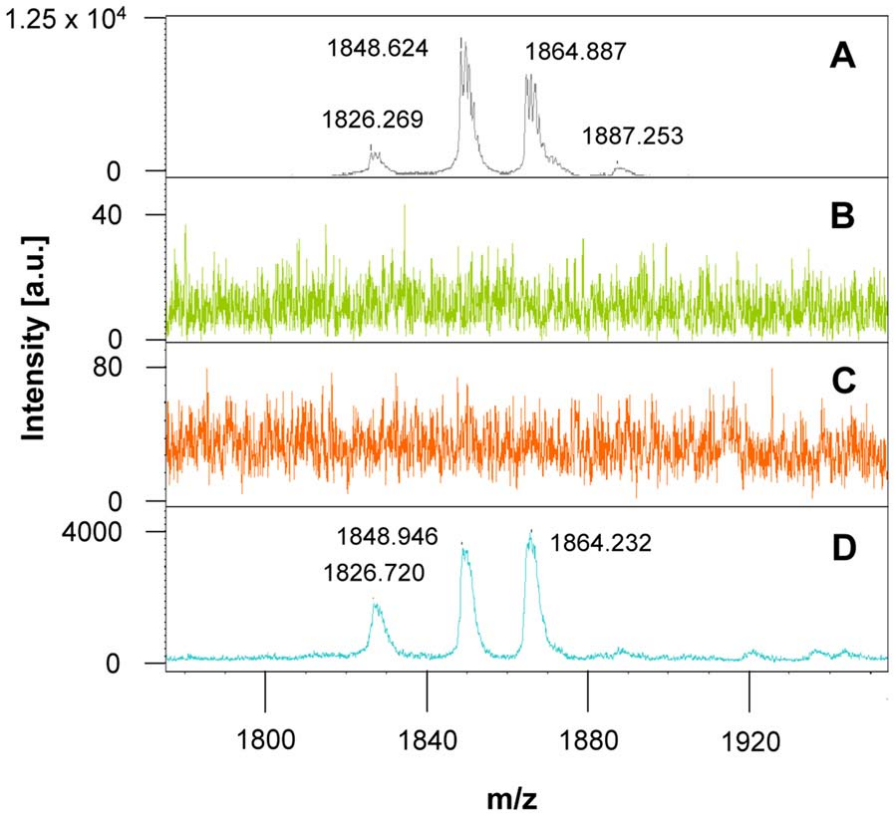
MALDI-TOF mass spectra of pure mersacidin and culture supernatants of *B. amyloliquefaciens* clones. Panel (**A**) shows pure mersacidin (control). The spectra of culture supernatants of *B. amyloliquefaciens* FZB42 (**B**) as well as of *B. amyloliquefaciens* mrs1 (**C**), which harbor only a part of the mersacidin gene cluster or miss the structural gene *mrsA*, respectively, show no mersacidin production. In contrast, the culture supernatant of *B. amyloliquefaciens* mrs1 pPAR1 (**D**) is characterized by the presence of the typical mersacidin-related mass signals [1826 Da: mersacidin + H, 1848 Da: mersacidin + Na and 1864 Da: mersacidin + K] which demonstrate the production of mersacidin by this strain.

In order to demonstrate the expression of mersacidin, MALDI-TOF analysis of the culture supernatant of *B. amyloliquefaciens* mrs1 pPAR1 was performed and spectra were compared to those of *B. amyloliquefaciens* FZB42 (wild type) as well as the mrs1 strain, missing *mrsA* (Fig. 2). The typical mersacidin masses [1826 Da: mersacidin + H, 1848 Da: mersacidin + Na and 1864 Da: mersacidin + K] were detected in the culture supernatant of the strain harboring pPAR1, indicating expression of fully modified mersacidin. In contrast, the spectra of *B. amyloliquefaciens* FZB42 and of *B. amyloliquefaciens* mrs1 did not show any mersacidin-related mass peaks.

On the other hand, in agar well diffusion assays with supernatants of cultures incubated in the absence of chloramphenicol, the inhibition zones of *B. amyloliquefaciens* mrs1 pPAR1 against *M. luteus*, *S. aureus* SG511 and *Bacillus megaterium* were not significantly larger than those produced by *B. amyloliquefaciens* FZB42 or *B. amyloliquefaciens* mrs1. The reason for this observation was that *B. amyloliquefaciens* FZB42 is able to produce an array of antimicrobial and antifungal substances including polyketides (bacillaene, difficidin, macrolactin), lipopeptides (surfactin, fengycin, bacillomycin D), two siderophores (bacillibactin, product of *nrs* cluster), the antimicrobial dipeptide bacilysin as well as the thiazole/oxazole containing antibiotic plantazolicin [14], [15]. Comparative MALDI-TOF spectra of *B. amyloliquefaciens* FZB42 and its mutant strains indeed indicated the presence of the lipopeptide surfactin and the antifungal compounds fengycin and bacillomycin D in culture supernatants of all tested *B. amyloliquefaciens* strains (data not shown). The activity of surfactin was also detected by hemolysis on Columbia blood agar plates. In conclusion, the antimicrobial activity of mersacidin was probably masked by the activity of surfactin in the agar well diffusion assays. The production of several antibacterial products by *Bacillus* strains is far from unusual. For example, secondary antibacterial compounds are also detected in the culture supernatants of the producer strain of mersacidin (Bierbaum, unpublished results) and even *B. subtilis* 168 - in spite of the mutation in *sfp* that inhibits production of the lipopeptides encoded in the genome [16] (surfactin [17] and plipastatin/fengycin [18]) - is still able to excrete at least four other antibacterial compounds, i. e. sublancin 168 [19], subtilosin A [20], bacilysocin [21] and bacilysin [22].

**Figure 3:**
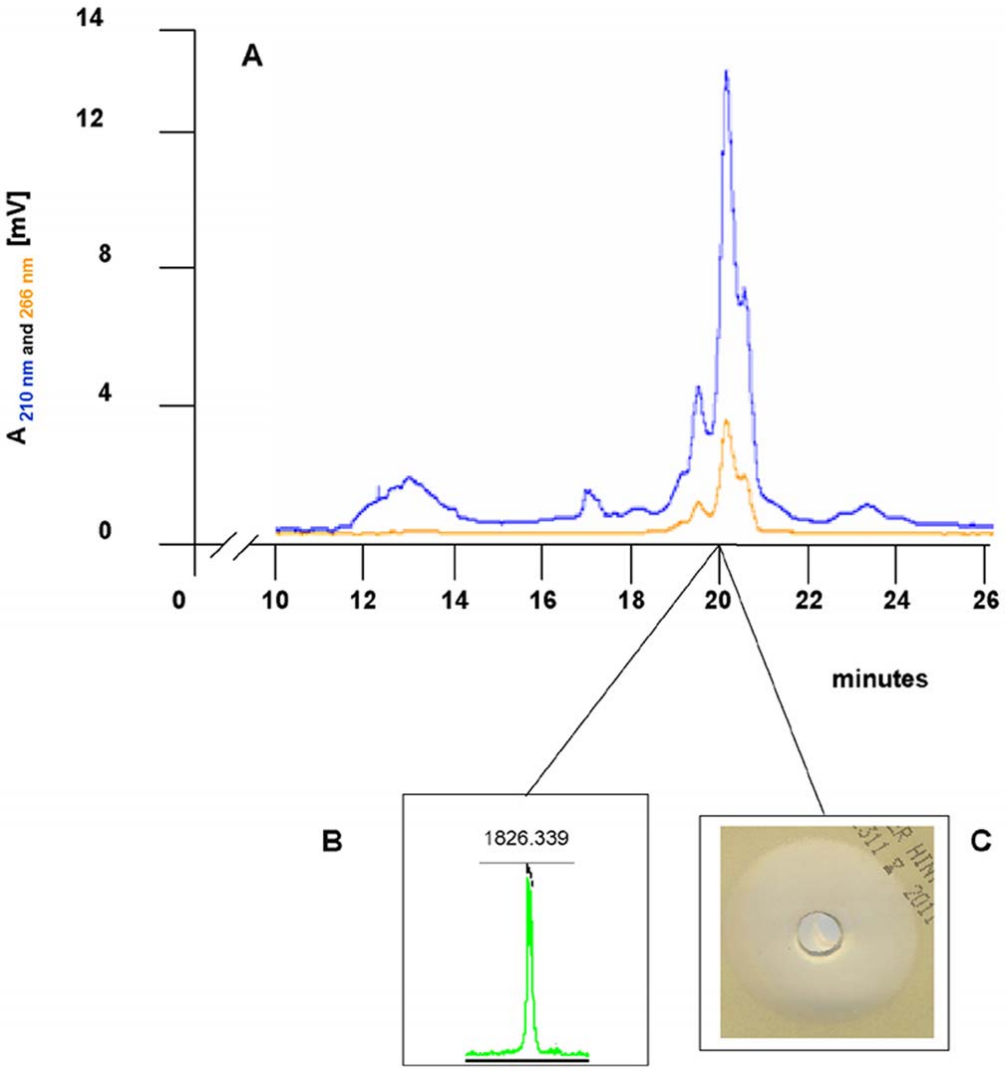
Purification of mersacidin from culture supernatant of *B. amyloliquefaciens* mrs1 pPAR1. The culture supernatant of *B. amyloliquefaciens* mrs1 pPAR1 was applied to a Poros RP-HPLC column and eluted in a gradient of 30 % to 42 % acetonitrile (containing 0.1 % TFA). Active fractions were pooled and lyophilized. The resulting lyophilizate was resuspended in 5 % acetonitrile and applied to a Nucleosil RP-C18 column (**A**). The antimicrobial activity of the fractions was assayed in agar well diffusion tests against *M. luteus* (diameter 3.2 cm) (**C**) and analyzed by MALDI-TOF (**B**). Active fractions eluted after 20 min in a gradient of 50 – 65 % acetonitrile (0.1 % TFA) and were characterized by the presence of a peak with 1826.339 Da representing mersacidin.

In order to demonstrate that the mersacidin produced by *B. amyloliquefaciens* mrs1 pPAR1 was correctly modified and showed antimicrobial activity, it was partially purified by two consecutive HPLC runs from cultures grown in the presence of chloramphenicol. The HPLC fractions that were active against *M. luteus* and that showed the typical adsorption spectrum of mersacidin were further analyzed by MALDI-TOF and showed the presence of the typical mass signals (Fig. 3). These results indicated *B. amyloliquefaciens* mrs1 pPAR1 had produced active and fully modified mersacidin.

### The producer strain of mersacidin belongs to the species *B. amyloliquefaciens*

Upon comparison of various DNA sequences (*yvnB*, *czcO*, *hpr*, *baeD*, *hemE*, and *comK*) obtained from the producer strain of mersacidin to the sequences of the *B. amyloliquefaciens* FZB42 genome in NCBI, a high similarity between both strains became obvious. Table 1 demonstrates that all sequences, including intergenic regions, showed about 98.5 % nucleotide sequence identity to those of *B. amyloliquefaciens* FZB42 and about 93.5 % identity to the sequences of the type strain *B. amyloliquefaciens* DSM 7^T^, whereas the identity to *B. subtilis* 168 was considerably lower (77.4 %).

**Table 1:** Results of discontiguous megablasts employing nucleotide sequences of *Bacillus* sp. HIL Y-85,54728.

gene	*B. amyloliquefaciens* FZB42		*B. amyloliquefaciens* DSM 7^T^		*B. subtilis* 168	
	identical bases/total bases	percent identity	identical bases/total bases	percent identity	identical bases/total bases	percent identity
*yvnB*	628/638	98.4	595/638	93.2	464/638	72.7
*czcO*	747/758	98.5	164/192	85.4	651/788	82.6
*baeD*	337/343	98.2	319/343	93.0	174/240	72.5
*yhaI, hpr*	768/779	98.5	746/779	95.7	632/782	80.8
*hemE*	217/220	98.6	216/220	98.1	167/212	78.7
*comK*	802/814	98.5	780/814	95.8	559/720	77.6
mean value		98.5		93.5		77.4

During BLAST searches, the genome sequence of *B. amyloliquefaciens* FZB42 always showed the highest similarity to the sequence of the producer strain. *B. amyloliquefaciens* DSM 7 scored second, with the exception of the *czcO* region which seems to be partially missing in this strain, followed by *B. subtilis* 168 in third position (with the single exception of *hemE*, here *B. subtilis* W23 scored third with 168/212 identical bases).

**Figure 4:**
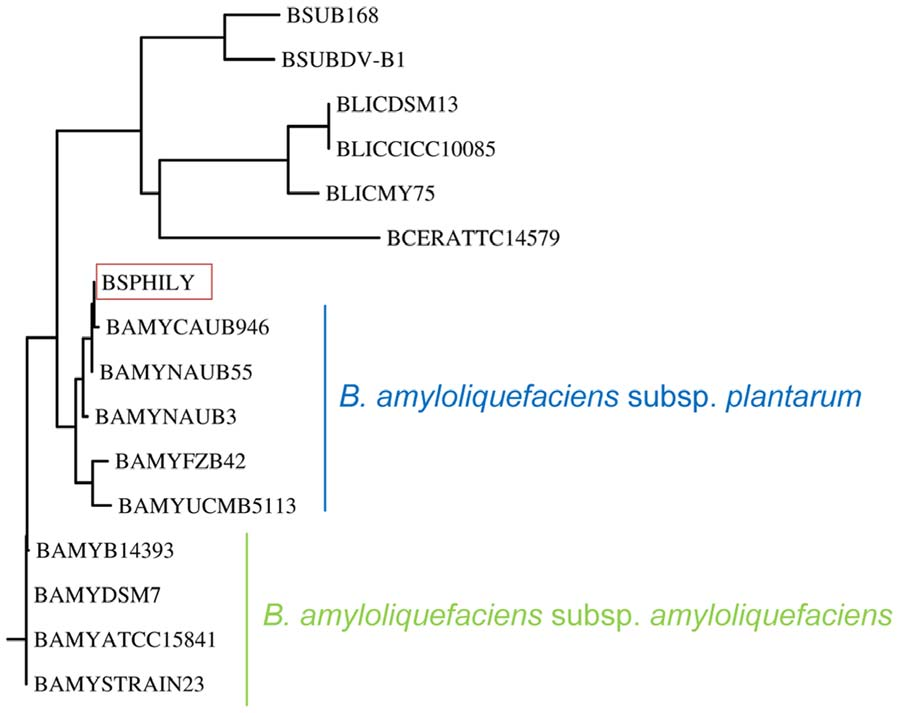
Phylogenetic tree based on the partial nucleotide sequence of the *gyrA* gene. The tree was calculated based on the *gyrA* nucleotide sequences of the mersacidin producer (BHILY, marked by a red box) and different members of the genus *Bacillus* (NCBI accession numbers in brackets) [*B. amyloliquefaciens*  =  BAMY strains: FZB42 (CP000560), CAUB946 (FN652789), S23 (FN652780), ATCC15841 (FN662838), DSM7 (FN597644), NAUB3 (FN652783), NAUB55 (FN652801), UCMB5113 (AY212974); *B. licheniformis*  =  BLIC strains: MY75 (EU073420), DSM13 (BLi00007), CICC10085 (GQ355995); *B. subtilis*  =  BSUB strains: 168 (BSU00070), DV1-B1 (EF134416) and *B. cereus*  =  BCER strain: ATCC14579 (BC0006)]. The mersacidin wild type producer is placed among the members of the subspecies *B. amyloliquefaciens* subsp. *plantarum*; it does not belong to the subspecies *amyloliquefaciens* that consists of strains closely related to the type strain *B. amyloliquefaciens* DSM 7^T^. It is also clearly not a member of the species *B. subtilis, B. cereus* or *B. licheniformis*.

The presence of *baeD* showed that *Bacillus* sp. HIL Y-85,54728 carries at least parts of the bacillaene gene cluster that was described for *B. amyloliquefaciens* FZB42 [13] and *B. amyloliquefaciens* DSM 7^T^ [23]. A biochemical identification test demonstrated that, in contrast to *B. subtilis* 168, *B. amyloliquefaciens* FZB42 and *Bacillus* sp. HIL Y-85,54728 both were able to metabolize xylose, lactose and starch and did not grow at 50°C nor in the presence of 10 % NaCl. Furthermore, both strains did not produce acid from trehalose and mannitol. The only difference between *B. amyloliquefaciens* FZB42 and *Bacillus* sp. HIL Y-85,54728 was the absence of gelatinase in the latter strain. 16S rRNA sequencing was performed with a PCR product that had been obtained using chromosomal DNA of *Bacillus* sp. HIL Y-85,54728 as a template. An NCBI BLAST search yielded a close similarity to *B. amyloliquefaciens* FZB42 16S rRNA (Table 2), indicating that *Bacillus* sp. HIL Y-85,54728 belongs to the species *B. amyloliquefaciens.* This was confirmed by the sequence of the gyrase gene *gyrA* (Fig. 4) and, therefore, we propose that the producer strain of mersacidin should be renamed “*B. amyloliquefaciens* HIL Y-85,54728”.

**Table 2:** Comparison of the sequences of the *Bacillus* sp. HIL Y-85,54728 16S rRNA genes with all *rrn* paralogs of *Bacillus amyloliquefaciens* DSM 7^T^, *Bacillus amyloliquefaciens* FZB42 and *Bacillus subtilis* 168.

strain/nucleotide position^a^	181	186	203	286	466	473	484
*B.* sp. HIL Y-85,54728	C/G^b^	C/T	G	A/G	G	A	C
*B. amyloliquefaciens* FZB42	G	C/T	G	A/G	G	A	C
*B. amyloliquefaciens* DSM 7^T^	C	T	A/G	G	G	A	C
*B. subtilis* 168	G	A	A	A	A	G	T

a The nucleotide positions correspond to [29]. All other bases are conserved between the four strains.

b The PCR product was directly sequenced, therefore, it contained all *rrn* paralogs and gave three mixed positions. An alignment of all seven paralogs of the *B. amyloliquefaciens* FZB42 16S rRNA with the sequence of the producer strain showed that two of the three mixed positions of *Bacillus* sp. HIL Y-85,54728 vary in *B. amyloliquefaciens* FZB42 in the same manner. The third mixed position (181) contained the C that is present in all *rrn* paralogs of *B. amyloliquefaciens* DSM 7^T^ and the G that is found in all paralogs of *B. amyloliquefaciens* FZB42.

*B. amyloliquefaciens* FZB42 was isolated from the plant rhizosphere and has recently been defined as the type strain of a group of growth-promoting plant associated *B. amyloliquefaciens* strains (*B. amyloliquefaciens* subsp. *plantarum*) [24]. In addition to their ability to colonize plant roots, the members of the *plantarum* subspecies are discriminated from the subspecies *B. amyloliquefaciens* subsp. *amyloliquefaciens* by differences in the *gyrA* and *cheA* nucleotide sequence, hydrolysis of cellulose and an increased ability to produce nonribosomal secondary metabolites like fengycin and difficin [24]. In a taxonomic tree that was calculated from the *gyrA* nucleotide sequences of the mersacidin producer and different members of the genus *Bacillus*, the producer strain is found in the cluster formed by the members of the *plantarum* subspecies, suggesting a close association between the mersacidin wild type producer and these strains (Fig. 4). The similarity was not confined to the *gyrA* gene but was also reflected by the higher overall nucleotide sequence identity (98.5 %) of the mersacidin wild type producer and *B. amyloliquefaciens* subsp. *plantarum* FZB42 in comparison to the value reached by *B. amyloliquefaciens* subsp. *amyloliquefaciens* DSM 7^T^ (93.5 %) (Table 1).

A distinguishing feature of the subspecies *B. amyloliquefaciens* subsp. *plantarum* is their ability to colonize *Arabidopsis* roots [24]. In fact, nearly all members of this subspecies were isolated from plants, plant roots or like *B. amyloliquefaciens* FZB42 from infested soil. The only exception is represented by strain UCMB5113 that was isolated from soil. In contrast, the producer strain of mersacidin originates from soil of a salt pan in Mulund, India, and unfortunately a plant association of the original isolate was not mentioned in the first report [1]. However, the strain did not grow in the presence of 10 % salt in the laboratory, indicating that the salt pan might not be its natural biotope and that its presence in the sample might rather be due to its ability to form long-lived spores.

In conclusion, we could show here that it is possible to produce mersacidin in *B. amyloliquefaciens* FZB42. The successful production of fully modified and active mersacidin by this strain provides an appropriate *in vivo* expression system for the construction and expression of mersacidin analogs. The vast array of antibacterial and antifungal compounds that is already excreted by this organism is thought to provide competitive advantage in the rhizosphere [25]. It also harbors genes which are nearly identical to the immunity genes of mersacidin and which will afford additional protection against competing strains that excrete this lantibiotic. The strategy employed here, i. e. to use an organism that already possesses the immunity genes of a lantibiotic for production of the same substance, proved successful and led to the production of active and fully modified mersacidin.

## Materials and Methods

### Strains, plasmids, culture conditions and media

All bacterial strains and plasmids used in this study are listed in table 3. Strains were stored as 50 % glycerol stocks at −80°C. *Bacillus* strains were cultured in tryptic soy broth (TSB, Oxoid, Wesel, Germany) or on tryptic soy agar. *Escherichia coli* strains were cultivated in LB. All cultures were maintained at 37°C. For genetically manipulated strains, antibiotics were added to the growth media (ampicillin, 40 µg/ml; erythromycin, 25 µg/ml; chloramphenicol, 20 µg/ml).

**Table 3:** Strains and plasmids used in this study.

Microorganism/plasmid	Function	Source/reference
*E. coli* K12 strains	Cloning hosts	
*B. subtilis* 168	Type strain of *B. subtilis*	ATCC 23857[30]
*B.* sp. HIL Y-85,54728	Wild type mersacidin producer strain, strain collection of Sanofi-Aventis (Frankfurt, Germany), no. FH 1658	[1]
*B.* sp. HIL Y-85,54728 Rec1	Mersacidin wild type producer deletion mutant: *mrsA* replaced by *ermB*, no mersacidin production	[2]
*B. amyloliquefaciens* FZB42	Wild type strain, carrying the 5′ part of the mersacidin gene cluster (*mrsKR2FGE*)	[13]
*B. amyloliquefaciens* mrs1	Mutant strain carrying the mersacidin gene cluster; *mrsA* is replaced by *ermB*	this study
*M. luteus* ATCC 4698	Indicator strain	ATCC 4698
*S. aureus* SG511	Indicator strain	[31]
*B. megaterium* KM	Indicator strain	ATCC 13632
pCU1	Shuttle vector	[32]
pPAR1	pCU1 harboring the promoter of *mrsA*, *mrsA* and the regulator gene *mrsR1*	[3]

### Purification of nucleic acids and sequencing

Genomic DNA was prepared using the PrestoSpinD Bug Kit according to the recommendations of the supplier (Molzym, Bremen, Germany). Plasmid DNA was isolated using the Gene-Jet™ Plasmid Miniprep Kit (Fermentas, St. Leon-Rot, Germany). All nucleic acids were analyzed by agarose gel electrophoresis and by spectrophotometry (Nanodrop Technologies, Wilmington, USA).

DNA sequencing was performed by Sequiserve (Vaterstetten, Germany) or Seqlab (Göttingen, Germany). Plasmid DNA and PCR products were dissolved in EB buffer (Qiagen, Hilden, Germany). Primers used for PCR analyses are listed in table 4. Sequencing primers were designed using the Primer3: WWW primer tool (http://biotools.umassmed.edu/bioapps/primer3_www.cgi).

**Table 4:** Primers used in this study.

Primer	Gene	Sequence	Source
16s1550.r	16sRNA genes (*rrn*), *B. amyloliquefaciens*	AAGGAGGTGATCCAGCCG	[27] modified
16s9.f	16sRNA genes (*rrn*), *B. amyloliquefaciens*	AGAGTT TGATCCTGGCTCAG	[27] modified
ermB665.r	*ermB*	CAATTTAAGTACCGTTACTTATGAGC	this study
fbaB283.r	*fbaB, B. amyloliquefaciens* FZB42	CTCCCGCATGACTGATATTCCTC	this study
gyrA_F	*gyrA, B. amyloliquefaciens* FZB42	CAGTCAGGAAATGCGTACGTCCTT	[28]
gyrA_R	*gyrA, B. amyloliquefaciens* FZB42	CAAGGTAATGCTCCAGGCATTGCT	[28]
FZBfor	*comK, B. amyloliquefaciens* FZB42	ATGGGGTCGAAGGTCATTGAG	this study
FZBrev	*comK, B. amyloliquefaciens* FZB42	CAGCTCCCGCAAAATAAAGTCG	this study
mrsE681.f	*mrsE,* mersacidin gene cluster	TGTCTCGGTCTCCTGGTTTACG	this study
mrsT2251.f	*mrsT,* mersacidin gene cluster	GGATAGACAGAAAGCTACGCTGC	this study

### Construction of the mersacidin producing *B. amyloliquefaciens* FZB42 mutants

The biosynthetic part of the mersacidin gene cluster was transferred to *B. amyloliquefaciens* FZB42 by competence transformation [26] using genomic DNA of the strain *B.* sp. HIL Y-85,54728 Rec1, which harbors a selection marker (*ermB* resistance cassette) instead of *mrsA*. Transformants were selected on TSA (0.3 µg/ml erythromycin) and were subsequently cultured on TSA containing erythromycin at a final concentration of 25 µg/ml. The transfer of the gene cluster was confirmed by PCR using primer combinations that anneal within *ermB* (ermB665.r), *mrsE* (mrsE681.f), *mrsT* (mrsT2251.f) and the downstream coding ORF *fbaB/iolJ* (fbaB283.r) (see Fig. 1). To exclude the possibility that these colonies might derive from germinated spores of *Bacillus* sp. HIL Y-85,54728 Rec1 that had not been eliminated during the gDNA preparation, differential PCRs were performed using the *comK* primers FZBfor and FZBrev. The 3′ ends of these primers anneal to single nucleotide polymorphisms of the sequence of *B. amyloliquefaciens* FZB42, but not to those of *B.* sp. HIL Y-85,54728. The resulting clone was named *B. amyloliquefaciens* mrs1.

For reconstitution of mersacidin production, the mersacidin structural gene (*mrsA*) was introduced *in trans* on the plasmid pPAR1 by competence transformation, yielding *B. amyloliquefaciens* mrs1 pPAR1. The presence of the plasmids as well as the plasmid integrity was analyzed by plasmid isolation and gel electrophoresis.

### Producer self-protection against mersacidin

To test the susceptibility of *B. amyloliquefaciens* FZB42, *B. amyloliquefaciens* mrs1, and *B. amyloliquefaciens* mrs1 pPAR1 to mersacidin, the minimal inhibitory concentration (MIC) of mersacidin was determined by arithmetic broth microdilution. Serial twofold dilutions of mersacidin were prepared in polystyrene round bottom microtiter plates (Greiner, Frickenhausen, Germany) using half concentrated Mueller Hinton II broth (Difco, Detroit, USA) containing 1 mM CaCl_2_. An inoculum of 5×10^5^ CFU/ml was employed in a final volume of 200 µl. The MICs were calculated from the lowest concentration of mersacidin resulting in the complete inhibition of visible bacterial growth after 16 hours of incubation at 37°C and compared to the MIC of the mersacidin producer *B.* sp. HIL Y-85,54728.

### Mersacidin production by *Bacillus amyloliquefaciens* mrs1 pPAR1

The production of mersacidin by *B. amyloliquefaciens* mrs1 harboring pPAR1 was assayed in 50 ml synthetic medium (2 x BPM) [2] in the presence of chloramphenicol. The cells were grown for 24 hours at 37°C with agitation (180 rpm). For further analysis the culture supernatant was sterilized by filtration and stored at −20°C. The detection of antimicrobial activity in 50 µl of culture supernatant and HPLC fractions was performed by agar well diffusion assays on Mueller-Hinton agar II plates (Difco, Detroit, USA) seeded with the indicator strains *Micrococcus luteus* ATCC 4698, *Bacillus megaterium* KM and *Staphylococcus aureus* SG511 in wells with a diameter of 7 mm. After incubation at 37°C overnight, the growth inhibition zones were measured.

In order to remove the chloramphenicol and other antibiotics excreted by *B. amyloliquefaciens* FZB42, 5 ml of the culture supernatant containing 0.1 % trifluoroacetic acid (TFA, Sigma-Aldrich, Taufkirchen, Germany) were applied to a Poros RP-HPLC-column (10R2, 10064.6 mm Perseptive Biosystems, Freiburg, Germany) and eluted in a gradient of 30 % to 42 % acetonitrile (containing 0.1 % TFA). The peaks were detected measuring the absorbance at 210 or 220 and 266 nm. The fractions were collected and assayed for the antimicrobial activity against *M. luteus* ATCC 4698 in agar well diffusion assays. The active fractions of 85 ml of culture supernatant were lyophilized and purified further using an RP C-18 column (Nucleosil-100-C18, 250×4.5 mm; Schambeck SFD GmbH, Bad Honnef, Germany) with a gradient of 50 to 65 % acetonitrile (containing 0.1 % TFA). For MALDI-TOF analysis (Bruker Biflex, Bruker Daltonics, Bremen, Germany) of culture supernatant and active HPLC fractions, 20 µl of each fraction were concentrated 1∶10 using a rotational Vacuum Concentrator (RVC 2–18, Christ, Osterode, Germany). Then, a 1 µl sample was mixed with 2 µl matrix (alpha-cyano-4-hydroxycinnamic acid in acetonitrile: 0.1 % TFA in water, 1∶3). The mixtures were spotted onto the MALDI target and air-dried. Mass spectra were measured in positive ion mode in the range of 500 to 4000 Da and analyzed by Flexanalysis 2.0 (Bruker Daltonics).

### Species identification of *B.* sp. HIL-Y-85,54728

The abilities of the wild type mersacidin producer strain, *B. amyloliquefaciens* FZB42 and *B. subtilis* 168 to ferment dextrose, maltose, lactose, sucrose, xylose, trehalose, mannitol and starch, to reduce sulfur and nitrate and to hydrolyze tryptophan and gelatin were compared using a conventional biochemical identification test. All inoculated tubes were incubated at 37°C for 24 h. Furthermore, growth at high salt concentrations (10 %) and high temperature (50°C) as well as motility were tested. The PCR product of the 16S rRNA genes was sequenced using the primers 16s9.f and 16s1550.r [27]. The gene coding for the gyrase subunit A (*gyrA*) was partially sequenced employing the primers gyrA_F and gyrA_R [28].

### Bioinformatic tools and nucleotide sequence accession numbers

The sequences of the chromosome of *B. subtilis* 168 and *B. amyloliquefaciens* FZB42 are deposited in the NCBI database CoreNucleotide under the accession numbers NC_000964 and NC_009725, respectively. The sequence of the mersacidin gene cluster has the accession number AJ250862. Further sequences of the mersacidin producer strain have been deposited at NCBI under the following accession numbers: *yvn*, JF519627; *czcO*, JF519628; *hpr*, JF519629; *baeD*, JF519630; *hemE*, JF519631; *comK*, JF519632; *gyrA*, JF519633.

Blasts were performed at the NCBI nucleotide website (http://blast.ncbi.nlm.nih.gov/Blast.cgi). ClustalW2 (http://www.ebi.ac.uk/Tools/msa/clustalw2/) and ClustalW 2.0.12 (http://mobyle.pasteur.fr/cgi-bin/portal.py#forms::clustalw-multialign) were employed for multi sequence alignments of *gyrA* and calculation of the phylogenetic tree.
